# Combined Treatment of MCF-7 Cells with AICAR and Methotrexate, Arrests Cell Cycle and Reverses Warburg Metabolism through AMP-Activated Protein Kinase (AMPK) and FOXO1

**DOI:** 10.1371/journal.pone.0150232

**Published:** 2016-02-26

**Authors:** Tamás Fodor, Magdolna Szántó, Omar Abdul-Rahman, Lilla Nagy, Ádám Dér, Borbála Kiss, Peter Bai

**Affiliations:** 1 Department of Medical Chemistry, Faculty of Medicine, University of Debrecen, Debrecen, H-4032, Hungary; 2 Department of Oncology, Section of Radiation Therapy, Faculty of Medicine, University of Debrecen, Debrecen, H-4032, Hungary; 3 Department of Dermatology, Faculty of Medicine, University of Debrecen, Debrecen, H-4032, Hungary; 4 MTA-DE Cell Biology and Signaling Research Group, Debrecen, H-4032, Hungary; 5 MTA-DE Lendület Laboratory of Cellular Metabolism, Debrecen, H-4032, Hungary; 6 Research Center for Molecular Medicine, University of Debrecen, Debrecen, H-4032, Hungary; Hungarian Academy of Sciences, HUNGARY

## Abstract

Cancer cells are characterized by metabolic alterations, namely, depressed mitochondrial oxidation, enhanced glycolysis and pentose phosphate shunt flux to support rapid cell growth, which is called the Warburg effect. In our study we assessed the metabolic consequences of a joint treatment of MCF-7 breast cancer cells with AICAR, an inducer of AMP-activated kinase (AMPK) jointly with methotrexate (MTX), a folate-analog antimetabolite that blunts *de novo* nucleotide synthesis. MCF7 cells, a model of breast cancer cells, were resistant to the individual application of AICAR or MTX, however combined treatment of AICAR and MTX reduced cell proliferation. Prolonged joint application of AICAR and MTX induced AMPK and consequently enhanced mitochondrial oxidation and reduced the rate of glycolysis. These metabolic changes suggest an anti-Warburg rearrangement of metabolism that led to the block of the G1/S and the G2/M transition slowing down cell cycle. The slowdown of cell proliferation was abolished when mitotropic transcription factors, PGC-1α, PGC-1β or FOXO1 were silenced. In human breast cancers higher expression of AMPKα and FOXO1 extended survival. AICAR and MTX exerts similar additive antiproliferative effect on other breast cancer cell lines, such as SKBR and 4T1 cells, too. Our data not only underline the importance of Warburg metabolism in breast cancer cells but nominate the AICAR+MTX combination as a potential cytostatic regime blunting Warburg metabolism. Furthermore, we suggest the targeting of AMPK and FOXO1 to combat breast cancer.

## Introduction

Cancer cells, among them, breast cancer cells undergo marked metabolic changes upon transformation that is called Warburg metabolism characterized by enhanced glycolysis and reduced mitochondrial oxidation [1]. Tumor cells are hypermetabolic and utilize external (glucose, glutamine) and internal (glycogen) substrates extensively [1–3]. Glucose degradation (both from external and internal source) through glycolysis is the major ATP source of tumor cells, therefore glycolytic rate is increased in tumors [3]. Furthermore, enhanced glycolysis boost the pentose-phosphate shunt that is vital for *de novo* nucleotide production [1]. Meanwhile, mitochondria, through expressing an alternative enzyme set utilizes glutamine to supply cells with vital intermediates (e.g. citrate for fatty acid synthesis) to support extensive rate of cell division [1]. Importantly, the G1/S checkpoint in cell cycle is under metabolic control, cell cycle is blocked if the metabolite pool, necessary for successful DNA replication, is missing [4,5].

The accommodation of cellular metabolism is regulated through an intricate web of stress sensors involving AMP-activated kinase (AMPK). AMPK is activated by increases in cellular ATP/AMP ratio [6]. Increases in the ATP/AMP ratio reflect decreases in cellular energy charge and in response to that, AMPK induces catabolic and, simultaneously, reduces anabolic processes [6]. AMPK is in intricate relations with other energy/metabolite sensor pathways (e.g. SIRT1, Akt, mTOR, PARPs, etc.) and act in a coordinate fashion with those [3,6–9]. AMPK induction leads to enhanced mitochondrial oxidation and mitochondrial biogenesis that had been shown to exert anti-Warburg and antiproliferative effects in lymphomas [10]. In our studies we utilized 5-Aminoimidazole-4-carboxamide 1-β-D-ribofuranoside (AICAR) to pharmacologically induce AMPK [11].

Breast cancer is one the most frequent tumor among women worldwide and despite extensive prevention programs, still represent a major cause of mortality. Treatment options involve surgical excision of the tumor tissue and chemotherapy [12]. Methotrexate (MTX) is not considered as a first-line agent in chemotherapy of breast cancer, it is used in combination with cyclophosphamide and 5-fluoro-uracil that is termed the CMF regime [12]. MTX primarily targets *de novo* nucleotide synthesis by interfering with the folate-dependent 1 carbon metabolism. Breast cancer cells are characterized by Warburg rearrangement [13–16], furthermore, attempts to revert Warburg metabolism (also called an anti-Warburg rearrangement of metabolism) support chemotherapy [17]. The apparent importance of Warburg metabolism in breast cancer cells suggested that AMPK activation and the following metabolic rearrangements may have antiproliferative effects and may act synergistically with methotrexate.

## Methods

### Chemicals

All chemicals were from Sigma-Aldrich if not stated otherwise. For the pharmacological activation of AMPK, AICAR was used (Sigma-Aldrich or Santa Cruz Biotech).

### Cell Culture

MCF-7 cells—a generous gift from Dr. Árpád Lányi (Department of Immunology, University of Debrecen, Debrecen, Hungary)—were maintained in MEM (Sigma-Aldrich), 10% fetal bovine serum (Sigma-Aldrich), 1% Penicillin/Streptomycin (Invitrogene), 2 mM L-Glutamine. MCF-7 cells were treated with 100 μM AICAR and 10 μM MTX and AICAR+MTX as indicated through one or six days. Cells were harvested for further analysis at day one and six. Control cells were cultured in the same media and treated with vehicle (PBS).

SKBR-3 cells—a generous gift from Dr. Péter Nagy (Department of Biophysics and Cell Biology, University of Debrecen, Debrecen, Hungary)—were maintained in MEM (Sigma-Aldrich), 10% fetal bovine serum (Sigma-Aldrich), 1% Penicillin/Streptomycin (Invitrogene), 2 mM L-Glutamine. SKBR-3 cells were treated with 100 μM AICAR and 10 μM MTX and AICAR+MTX as indicated through three days. Cells were harvested for further analysis at day three. Control cells were cultured in the same media and treated with vehicle (PBS).

4T1 cells—a generous gift from Dr. Ferenc Gallyas Jr. (Department of Biochemistry, University of Pécs, Pécs, Hungary)—were maintained in MEM (Sigma-Aldrich), 10% fetal bovine serum (Sigma-Aldrich), 1% Penicillin/Streptomycin (Invitrogene), 2 mM L-Glutamine. 4T1 cells were treated with 300 μM AICAR, 7.8 nM MTX and 300 μM AICAR+7.8 nM MTX as indicated through two days. Cells were harvested for further analysis at day two. Control cells were cultured in the same media and treated with vehicle (PBS).

SAOS cells were cultured in DMEM medium(SIGMA Aldrich) supplemented with 10% foetal bovine serum (FBS), 2mM L-glutamine, and 0,5% penicillin/streptomycin in humidified atmosphere 95%air-5% CO2 at 37C. Cell were passaged in every third day by Trypsin/EDTA (SIGMA Aldrich).

WM35 cells were maintained in RPMI-1640 medium supplemented with 10%foetal bovine serum (FBS), 2mM L-glutamine, and 0,5% penicillin/streptomycin in humidified atmosphere 95%air-5% CO2 at 37C. Cell were passaged in every third day by Trypsin/EDTA (SIGMA Aldrich).

The cell lines used in the study are available from European Collection of Authenticated Cell Cultures (*ECACC*).

### Cell cycle analysis

Cell cycle analysis was performed as described in [18]. MCF-7 cells were seeded in 6-well plate (10 000 cells/well). Cell cycle analysis of AICAR, MTX and AICAR+MTX treated cells were carried out following fixation in 70% ethanol and staining with 50 μg/ml propidium iodide (PI) using FACSCalibur. The acquired data were processed with the BD CellQuest^™^ Pro. software (Beckton Dickinson, Mountain View, CA).

### Sulphorhodamine B assay

In order to detect growth inhibition sulphoradamine B (SRB) assay that measures changes in total protein content was applied. Cells were seeded in 96-well plate (3000–5000 cells/well). Cells were grown in the presence of vehicle, AICAR, MTX, AICAR+MTX for the time indicated (MCF-7 cells– 6 days, SKBR-3 cells– 3 days, 4T1 cells– 2 days, SAOS cells– 6 days, WM35–6 days). Cells were checked in an invert microscope every day. At the end of the treatment, after fixation in situ by 50% trichloroacetic acid (TCA) cell were stained with sulforhodamine B (SRB) solution (0.4% in 1% acetic acid). Unbound dye was removed by washing with 1% acetic acid. Bound stain was solubilized with 10 mM TRIS base. Absorbance was read on an automated plate reader (Thermo Labsystems Multiskan MS) at 540 nm.

### Cell death assays

Caspase and propidium-iodide uptake assays were performed as described in [19].

### SDS-PAGE and Western Blotting

Sample preparation, protein separation, transfer and immunolabelling was performed as in [20]. Briefly, cells were lysed in RIPA buffer (50 mM Tris, 150 mM NaCl, 10% SDS, 1% Nonidet P-40, 1 mM Na_3_VO_3_, and 1 mM NaF, 0, 5% sodium deoxycolate, 1 mM phenylmethylsulfonyl fluoride, protease inhibitor mixture, pH 8.0). Proteins were separated by sodium dodecyl sulfate-polyacrylamide gel electrophoresis (SDS-PAGE) on 10% acrylamide gels and blotted onto nitrocellulose membranes. After blocking in 5% (w/v) non-fat dry milk, the membranes were washed with 1X TWEEN-TBS and incubated with primary antibodies for overnight at 4°C. For the detection of AMPK activity phospho-acetylCoA carboxylase (pACC) (1:500), (Cell Signaling) was used. Actin was detected using a rabbit polyclonal antibody (Sigma, 1:5000). The secondary antibody was IgG peroxidase HRP conjugate (Sigma, 1:2000). Bands were visualized by enhanced chemiluminescence reaction (West Pico ECL Kit, Thermo Scientific).

### Measurement of mitochondrial membrane potential

Mitochondrial membrane potential was determined by (3,3′-dihexyloxacarbocyanine iodide) (DioC6) staining as described in [20]. MCF-7 cells were seeded in 96-well plate (25 000 cells/well). After one or six day treatment, cells were harvested by adding trypsin/EDTA, and the detached cells were stained with 40 nM DioC6 for 30 min) then washed with phosphate-buffered saline (PBS). Cells were subjected to flow cytometric analysis (FACSCalibur, BD Biosciences) with 20,000 events collected for each sample; each measurement point was repeated in 3 parallel replicates. Control cells were treated with 10 μM Carbonyl cyanide-4-(trifluoromethoxy)phenylhydrazone (FCCP) to dissipate mitochondrial membrane potential. The value measured in the FCCP-treated cells were subtracted from all groups. The FCCP-corrected values were displayed and were used for statistical analysis.

### RNA isolation, reverse transcription and QPCR

Total RNA was prepared using TRIzol reagent (Invitrogen) according to the manufacturer's instructions. Two micrograms of RNA were used for reverse transcription (High Capacity cDNA Reverse Transcription Kit, Applied Biosystems, Foster City, CA, USA). Diluted cDNA was used for reverse transcription-coupled quantitative PCR (RT-qPCR). The qPCR reactions were performed with the qPCRBIO SyGreen Lo-ROX Supermix (PCR Biosystems) using a Light-Cycler 480 system (Roche Applied Science). Gene expression was normalized to the geometric mean of human 36B4, 18S, and cyclophyllin values or for murine samples to the geometric mean of human 36B4, GAPDH, and cyclophyllin values as in [21]. Primers are listed in Table 1.

**Table 1:** List of primers.

	Forward
ACO2 (human)	F: CCA GAG ACC GAC TAC CTG ACG; R. CCA CTT GTC AAA AGG CTC CAG
Aktin (human)	F: GAC CCA GAT CAT GTT TGA GAC C; R: CAT CAC GAT GCC AGT GGT AC
AMPK (human)	F: CAT GAA GAG GGC CAC AAT CAA; R: GCC AAA GGA TCC TGG TGA TTT
ATP5g1 (human)	F: CTA AAC AGC CTT CCT ACA GCA ACT T; R: TGA ACC AGC CAC ACC AAC TGT
ATP5g1 (murine)	F: GCT GCT TGA GAG ATG GGT TC; R: AGT TGG TGT GGC ATC A
Cyclophillin A (human)	F: GTC TCC TTT GAG CTG TTT GCA GAC; R: CTT GCC ACC AGT GCC ATT ATG
Cyclophillin A (murine)	F: TGG AGA GCA CCA AGA CAG ACA; R: TGC CGG AGT CGA CAA TGAT
FOXO1 (human)	F: GTT CAT TGA GCG CTT AGA CTG; R: AAG TGT AAC CTG CTC ACT AAC CC
FOXO1 (murine)	F: AAG GAT AAG GGC GAC AGC AA; R: TCC ACC AAG AAC TCT TTC CA
Fumarase (human)	F: CTC GTT TTG GCC TCC GAA CG; R: TAA CTG GGG TTG GCA TGC GT
Fumarase (murine)	F: CGC AGG TCA TCA AAA TTG GGC G; R: GGG CAG TGA CAA AAG GCA AAC C
GAPDH (murine)	F: CAA GGT CAT CCA TGA CAA CTT TG; R: GGC CAT CCA CAG TCT TCT GG
IDH2 (human)	F: GGT GGA GAT GGA TGG TGA TGA; R: GTG ATG GTG GCA CAC TTG ACT
IDH2 (murine)	F: CGC CAT TAC CGA GAA CAC CAG A; R. GTG GTG TTC AGG AAG TGC TCG T
PGC-1α (human)	F. AGA ATT GGC TTA TGG ATG TAC AGG; R: TTT GTT GAT CAT TTC CAG CAA TAA T
PGC-1β (human)	F: GTG CTG ACA AGA AAT AGG AGA GG; R: CTC TTC TGA ATT GGA ATC GTA GTC
18s (human)	F: TCG AGG CCC TGT AAT TGG AAT; R: TCC CAA GAT CCA ACT ACG AGC TT
36B4 (human)	F: CCA TTG AAA TCC TGA GTG ATG TG; R: GTC GAA CAC CTG CTG GAT GAC
36B4 (murine)	F: AGA TTC GGG ATA TGC TGT TGG; R: AAA GCC TGG AAG AAG GAG GTC

### Oxygen consumption

Oxygen consumption was measured using an XF96 oxymeter (Seahorse Biosciences, North Billerica, MA, USA) similarly to [20]. MCF-7 cells were seeded in 96-well assay plates (~2000 cell/well) and were treated for AICAR, MTX and AICAR+MTX for the time indicated. Oxygen consumption rate (OCR, reflecting mitochondrial oxidation) and changes in pH, extracellular acidification rate (ECAR, reflecting glycolysis) were recorded every 30 min to follow AICAR and MTX effect. Data were normalized to protein content and normalized readings were displayed.

### Constructs, transfections

For silencing assays the pSUPER RNAi system was used similarly as in [22]. To create a small hairpin RNA (shRNA)-expressing construct, double stranded DNA oligonucleotides were cloned into the pSuper vector. The oligonucleotides (containing the siRNAsequence) were annealed in annealing buffer (150 mM NaCl, 1 mM EDTA, 50 mM Hepes, pH 8.0). The resulting duplexes carried BglII and HindIII sites and were cloned into pSuper using these sites, resulting a ready to transfect mammalian expression vector that directs intracellular synthesis of shRNA-like transcripts. Transfections were performed each day throghout the six days treatment using polyethylenimine (PEI) as a transfection reagent. Sequences of oligonucleotides are listed in Table 2.

**Table 2:** Sequence of DNA oligonucleotides used to generate pSuper constructs. The interfering sequences are in bold.

Name	Sequence (5’-3’)	Structure
siPGC1α-1 sense	GATCCCC**AAGACGGATTGCCCTCATTTG**TTCAAGAGA**CAAATGAGGGCAATCCGTCTT**TTTTTA	BglII /**sense**/loop/**antisense**/T(5)/HindII
siPGC1α-1 antisense	AGCTTAAAAA**AAGACGGATTGCCCTCATTTG**TCTCTTGAA**CAAATGAGGGCAATCCGTCTT**GGG	HindIII/T(5)/**antisense**/loop/**sense**/BglII
siPGC1α-2 sense	GATCCCC**GACTATTGCCAGTCAATTAAT**TTCAAGAGA**ATTAATTGACTGGCAATAGTC**TTTTTA	BglII /**sense**/loop/**antisense**/T(5)/HindII
siPGC1α-2 antisense	AGCTTAAAAA**GACTATTGCCAGTCAATTAAT**TCTCTTGAA**ATTAATTGACTGGCAATAGTC**GGG	HindIII/T(5)/**antisense**/loop/**sense**/BglII
siPGC1β sense	GATCCCC**AGCAACTCTATGCTGACTTTC**TTCAAGAGA**GAAAGTCAGCATAGAGTTGCT**TTTTTA	BglII /**sense**/loop/**antisense**/T(5)/HindII
siPGC1β antisense	AGCTTAAAAA**AGCAACTCTATGCTGACTTTC**TCTCTTGAA**GAAAGTCAGCATAGAGTTGCT**GGG	HindIII/T(5)/**antisense**/loop/**sense**/BglII
FOXO1 sense	GATCCCC**CAGGACAATAAGTCGAGTTAT**TTCAAGAGA**ATAACTCGACTTATTGTCCTG**TTTTTA	BglII /**sense**/loop/**antisense**/T(5)/HindII
FOXO1 antisense	AGCTTAAAAA**CAGGACAATAAGTCGAGTTAT**TCTCTTGAA**ATAACTCGACTTATTGTCCTG**GGG	HindIII/T(5)/**antisense**/loop/**sense**/BglII
AMPKα1 sense	GATCCCC**GTTGCCTACCATCTCATAATA**TTCAAGAGA**TATTATGAGATGGTAGGCAAC**TTTTTA	BglII /**sense**/loop/**antisense**/T(5)/HindII
AMPKα1 antisense	AGCTTAAAAA**GTTGCCTACCATCTCATAATA**TCTCTTGAA**TATTATGAGATGGTAGGCAAC**GGG	HindIII/T(5)/**antisense**/loop/**sense**/BglII

### Database screening

The effect of AMPKα1, FOXO1 and PGC-1α expression on breast cancer survival was assessed through the Kaplan-Meier plotter database (http://kmplot.com/analysis/ [23]) including those patients, where ER status was derived from gene expression data. Overall survival rates were analyzed.

### Statistical analysis

Significance was analyzed by Student’s *t* test, for multiple comparisons ANOVA test was applied. Error bars represent ± S.E., unless noted otherwise.

## Results

### The joint application of AICAR and MTX inhibits MCF-7 proliferation, but does not lead to cell death

**Fig 1:**
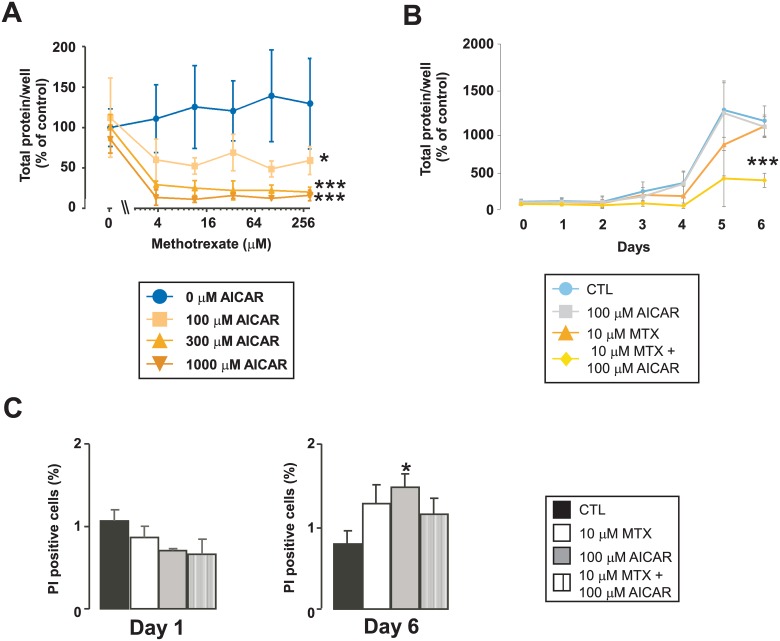
Combined treatment of AICAR and MTX decrease MCF-7 proliferation. **(A)** MCF-7 cells were seeded on 96-well culture plate at 3000–4000 cells/well and were treated with MTX and AICAR as indicated (n = 4 for each measurement point). After 6 days in culture total protein was determined using SRB assay. A typical experimental result is shown. **(B)** MCF-7 cells were seeded on 96-well culture plate at 3000–4000 cells/well and were treated with MTX and AICAR as indicated (n = 4 for each measurement point). Seven parallel plates were made and started on the same day. Each day a plate was fixed and at the end of the experiment total protein was determined using SRB in all plates. The measurement point day 0 represent the total protein before the beginning of treatment. A typical experimental result is shown. **(C)** MCF-7 cells were seeded on 6-well culture plate at 90 000–100 000 cells/well. Cells were treated with 100 μM AICAR and 10 μM MTX for 1 and 6 days (n = 3 for each measurement point). Dead cells were stained using propidium iodine, cells were harvested by tripsinization and were analyzed on a FacsCalibur flow cytometer. A typical experimental result is shown. * and *** indicate statistically significant difference between control and treated cells at p<0.05 and p<0.001, respectively. All abbreviations are in the text.

We began our investigations with assessing the sensitivity of MCF-7 cells (a cellular model of invasive ductal breast carcinoma) to AICAR and MTX. First, cells were treated with MTX (3–300 μM) or AICAR treatment (100–1000 μM) or in combination (Fig 1A). Cell proliferation was assessed through an invert microscope and on the sixth day of the assay total protein—that corresponds to cell number—was assessed using SRB assay. When cells were treated with MTX or AICAR alone we did not observe decreases in total protein content, however the combination of AICAR and MTX resulted in a marked decrease of total protein (Fig 1A). On day 6 10 μM MTX + 100 μM AICAR combination (abbreviated as AICAR+MTX) made a 50% reduction in cell numbers; these AICAR and MTX concentrations were used in the subsequent experiments.

As next step we made a time course experiment (Fig 1B). The effectiveness of AICAR+MTX combination became evident on the third day of treatment and turned statistically significant on day 6. To assess the dynamics of biochemical changes we made all measurements at an early time point (1 day post treatment, short treatment) and late time point (6 days post treatment, prolonged treatment).

An obvious reason for reduced total protein upon AICAR+MTX treatment could be cell death. Importantly, we did not detect major increase in PI positive cells neither on day 1, nor on day 6 (Fig 1C) or increases in caspase activity (data not shown), suggesting that increased cell death is not the cause of reduced cell numbers, it is due to rather lower proliferation rate.

### The joint application of AICAR and MTX induces AMPK that reverts Warburg metabolism

**Fig 2:**
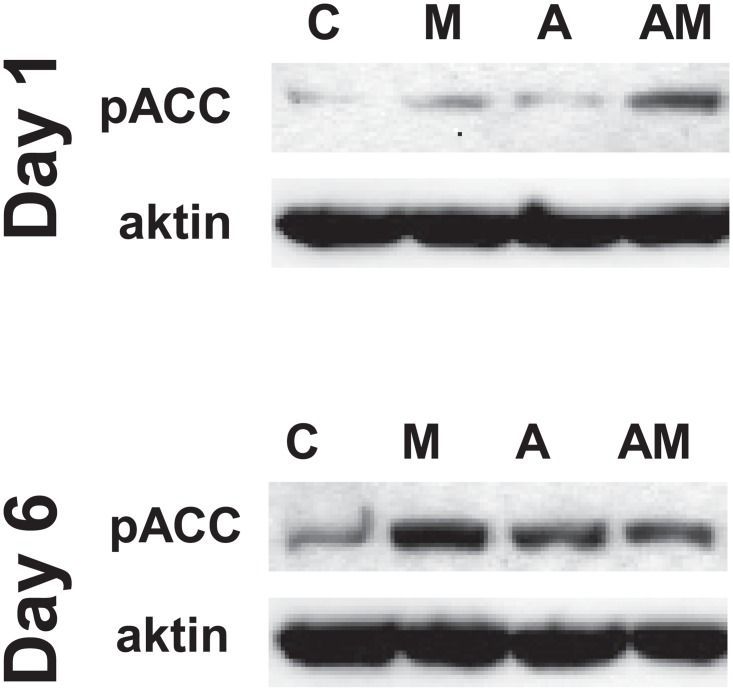
AMPK activation upon AICAR+MTX treatment. Activation of AMPK was determined by Western blot analysis of acetyl-CoA carboxylase phosphorylation (pACC) on day 1 and day 6. Actin was used as loading control. C: control; M: MTX; A: AICAR; AM: AICAR+MTX. A typical experimental result is shown.

Slowdown of proliferation may be the sign of an anti-Warburg rearrangement of metabolism. First, we tested that AICAR indeed activates AMPK by monitoring the phosphorylation of acetyl-CoA carboxylase (pACC). On day 1, similarly to the results on proliferation (Fig 1C), AICAR alone did not induce AMPK, only when used in combination with MTX. Later, on day 6, MTX, AICAR and MTX+AICAR robustly induced AMPK (Fig 2).

**Fig 3:**
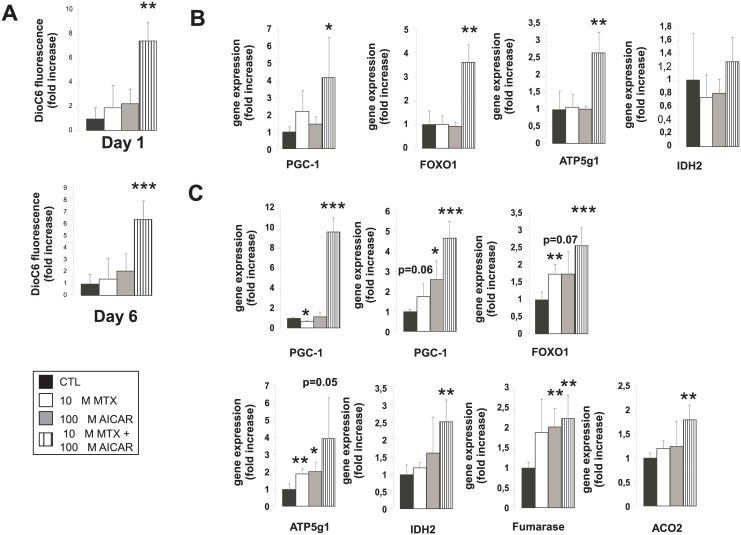
Activation of mitochondrial oxidation upon treatment with AICAR + MTX. **(A)** Mitochondrial membrane potential was measured by DiOC6 staining. MCF-7 cells were seeded in 96-well culture plate at 25 000 cells/well and were treated with MTX and/or AICAR as indicated (n = 4 for each measurement point). On day 1 and day 6 cell were loaded with DioC6, cells were harvested by tripsinization and were analyzed on a FacsCalibur flow cytometer. Mean of fluorescence intensity of 4 parallel samples were averaged and depicted. A typical experimental result is shown. **(B-C)** The expression of a set of gene were analyzed using RT-qPCR on (C) day 1 and (D) day 6 in MCF-7 cells treated with MTX and/or AICAR (n = 4/4/4/4) as indicated. Bars represent fold changes relative to control samples. All gene abbreviations are listed in the text. A typical experimental result is shown. *, ** and *** indicate statistically significant difference between control and treated cells at p<0.05, p<0.01 and p<0.001, respectively.

AMPK activation was translated into mitochondrial activation indicated by higher DioC6 fluorescence suggesting increases in mitochondrial membrane potential (Fig 3A). On day 1 we observed the induction of peroxisome proliferator activated receptor gamma coactivator (PGC)-1α and forkhead transcription factor-1 (FOXO1) upon AICAR+MTX treatment (Fig 3B). In line with these, the expression of ATP5g1, a subunit of ATP synthase and isocitrate dehydrogenase-2 (IDH2), a marker of tricarboxylic acid (TCA) cycle (Fig 3B) was induced slightly suggesting that higher expression of PGC-1α and FOXO1 was translated into gene expression programs supporting mitochondrial activity.

The expression of PGC-1α and FOXO1 further enhanced by day 6, furthermore, PGC-1β, another key mitotropic regulator [24–26] boosted upon AICAR+MTX treatment (Fig 3C). Consequently, on day 6 enhanced expression of ATP5g1 and IDH2 in the AICAR+MTX treated cells was exacerbated, moreover, other TCA cycle enzymes, fumarase and aconitase-2 (ACO2) were also induced (Fig 3C).

**Fig 4:**
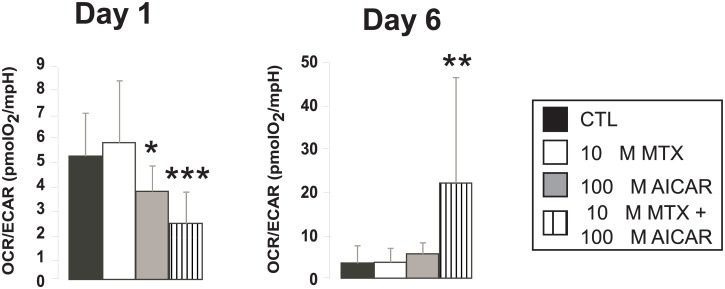
Changes in the ratio of glycolysis and mitochondrial oxidation upon treatment with AICAR + MTX. Rate of mitochondrial oxygen consumption and glycolysis were assayed by Seahorse XF96 analyzer on day 1 and day 6 as described in Materials and Methods (n = 23/23/23/23). The OCR/ECAR ratio was depicted that reflects the ratio of mitochondrial oxidation and glycolytic flux. *OCR*-oxygen consumption rate; *ECAR*-extracellular acidification rate. A representative result is shown. *, ** and *** indicate statistically significant difference between control and treated cells at p<0.05, p<0.01 and p<0.001, respectively.

As anti-Warburg rearrangement of metabolism leads to the suppression of glycolysis [1], we compared the rate of glycolysis and mitochondrial oxygen consumption using the Seahorse XF96 instrument. Interestingly, on day 1 in AICAR+MTX treated cells glycolysis was induced (Fig 4), evidenced by decreases in the ratio of oxygen consumption rate (OCR) / extracellular acidification rate (ECAR). In contrast to that, on day 6 OCR/ECAR ratio increased in AICAR+MTX pointing out increased mitochondrial activity and lower dependence of cells on glycolysis (Fig 4).

Taken together, treating MCF-7 cells with AICAR+MTX at early time point (i.e. day 1) moderately induces mitochondrial metabolism, but supports glycolysis—paradoxically bringing about rather pro-Warburg changes. However, protracted (i.e. day 6) AICAR+MTX treatment largely induces mitochondrial oxidation and suppresses glycolysis setting off true anti-Warburg changes.

### AICAR+MTX treatment leads to G1/S and G2/M blockade

**Fig 5:**
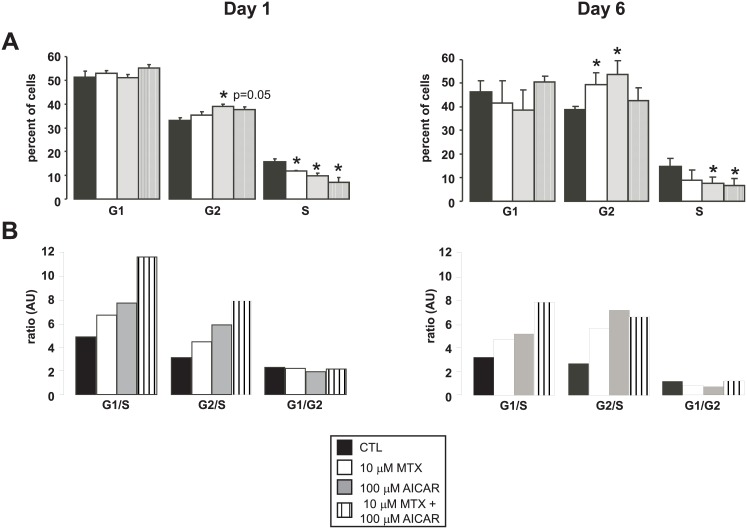
AICAR+MTX treatment blocks the cell cycle. **(A)** MCF-7 cells were seeded in 6-well plate at 10 000 cells/well (n = 6/6/6/6). Cells were treated with AICAR and/or MTX for the time indicated. Cells were fixed, permeabilized and charged with propidium iodide. DNA content was analyzed by flow cytometry. The data on day 1 is the average of 3 parallel experiments, while the data for day 6 is the average of 2 parallel experiments. **(B)** The data on panel A was further analyzed and the ratio between the different stages were calculated and depicted. The data derived from fig 5A. * indicate statistically significant difference between control and treated cells at p<0.05.

G1/S transition is under metabolic control: if cells do not possess the necessary substrates for successful replication cell cycle, G1 to S transition is blocked [5]. Our previous data supported an anti-Warburg rearrangement of cellular metabolism upon AICAR+MTX treatment, therefore we set out to analyze changes in cell cycle. Short term AICAR+MTX treatment (day 1) reduced the number of cells in S and slightly elevated the number of cells in G2 (Fig 5). When looking at the ratios between different cell cycle phases, it is apparent that G1/S and G2/S ratios increase, while G1/G2 ratio remains constant suggesting G2/S and probably G1/S block. Prolonged AICAR+MTX treatment (day 6) showed similar changes, increased proportion of G2 cells with decreased number of cells in S phase (Fig 5) and increases in the proportions of cells in G1 and G2 as compared to the number of cells in S—signs of simultaneous G1/S and G2/M block in the cell cycle.

### Combined treatment of SKBR-3 and 4T1 cells with AICAR and MTX reduces cell proliferation rate in an additive fashion

**Fig 6:**
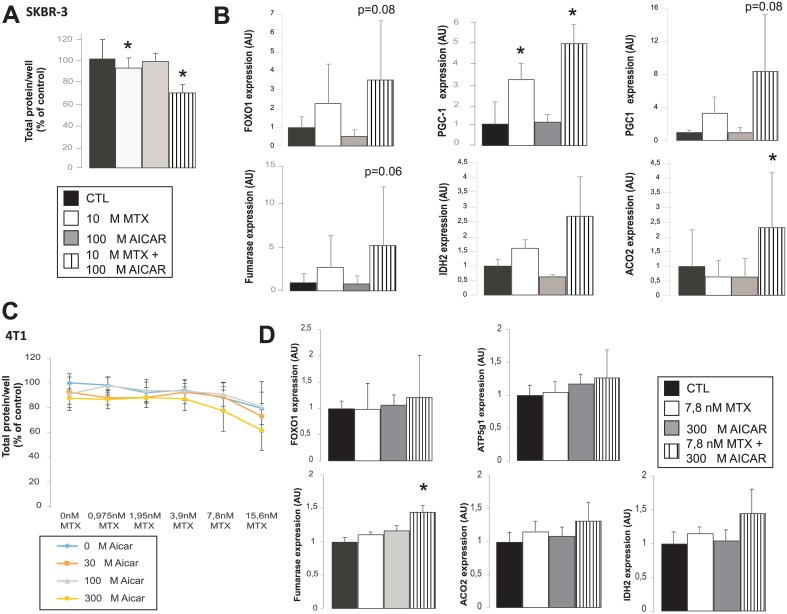
Combined treatment of SKBR-3 and 4T1 cells with AICAR and MTX reduces cell proliferation rate in an additive fashion. **(A)** SKBR-3 cells were seeded on 96-well culture plate at 3000–4000 cells/well and were treated with MTX and AICAR as indicated (n = 4 for each measurement point). After 3 days in culture total protein was determined using SRB assay. The data is the average of 3 parallel experiments. **(B)** The expression of a set of gene were analyzed using RT-qPCR on day 3 in SKBR-3 cells treated with MTX and/or AICAR (n = 6/6/6/6) as indicated. Bars represent fold changes relative to control samples. All gene abbreviations are listed in the text. A typical experimental result is shown. **(C)** 4T1 cells were seeded on 96-well culture plate at 3000–4000 cells/well and were treated with MTX and AICAR as indicated (n = 4 for each measurement point). After 2 days in culture total protein was determined using sulphorhodamine B. The data is the average of 3 parallel experiments. **(D)** The expression of a set of gene were analyzed using RT-qPCR on day 2 in 4T1 cells treated with MTX and/or AICAR (n = 6/6/6/6) as indicated. Bars represent fold changes relative to control samples. All gene abbreviations are listed in the text. A typical experimental result is shown. * indicate statistically significant difference between control and treated cells at p<0.05.

All previous data were obtained on one cell line, therefore we verified our findings on two other breast cancer cell lines, the human SKBR-3 and the murine 4T1. SKBR-3 cells, similarly to MCF-7, were not sensitive to MTX and AICAR when administered alone, while the AICAR+MTX combination slowed down cellular proliferation (Fig 6A) that coincided with increases in the expression of several markers of mitochondrial oxidation (FOXO1, PGC-1α, PGC-1β, fumarase, IDH2 and ACO2) that are the same as in the case of MCF-7 (Fig 6B).

4T1 cells were very sensitive to MTX, roughly thousand fold less MTX was already antiproliferative in 4T1 cells as compared to MCF-7 or SKBR-3 (Fig 6C). Despite the changes in sensitivity we did observe a slightly enhanced antiproliferative effect of the 300 μM AICAR + 7.8 nM MTX or 300 μM AICAR + 15.6 nM MTX combination as compared to the individual components alone (Fig 6C). Similarly to MCF-7 and SKBR-3 300 μM AICAR + 7.8 nM MTX treatment induced slightly the expression of FOXO1, ATP5g1, fumarase and IDH2 (Fig 6D). Taken together, the additive effect of AICAR+MTX treatment is not specific for MCF-7 but it acts similarly in other breast cancer cell lines as well. We tested two other cell lines, WM35 that is a model for melanoma and SAOS that is a model for osteosarcoma; none of them was susceptible to the MTX+AICAR combination (data not shown).

### The inhibitory properties of the AICAR+MTX treatment can be reverted by the silencing of mitotropic transcription factors

AICAR+MTX treatment brought about anti-Warburg alterations in metabolism and led to G1/S and G2/M block in cell cycle. Enhanced expression of PGC-1α, PGC-1β and FOXO1 (Fig 3D) correlates with enhanced mitochondrial activity and the slowdown of proliferation suggesting central role for these proteins in the antiproliferative effect of AICAR+MTX treatment. We prepared shRNA expressing constructs targeting these proteins in order to assess their possible role.

**Fig 7:**
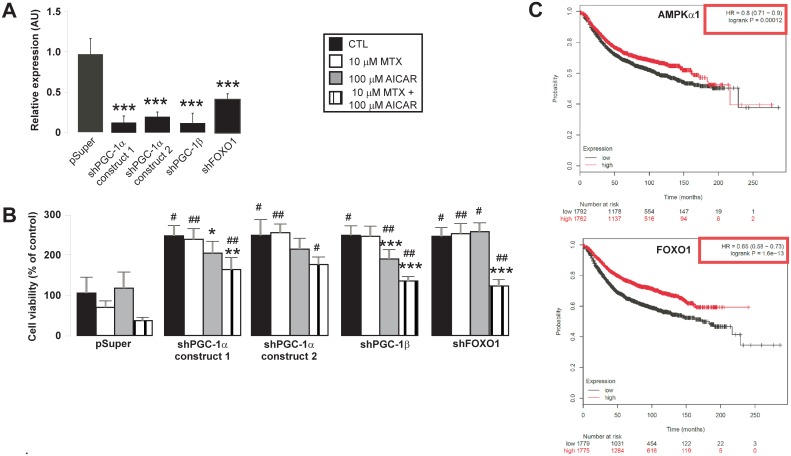
Mitotropic transcription factors have key role in the cytostatic effect of AICAR + MTX treatment. **(A)** The genes indicated were silenced in MCF-7 cells using small interfering RNAs, targeting the indicated genes, were cloned into the pSuper vector. Control cells were transfected with the parent plasmid, pSuper. Cells were harvested 1 day post-transfection (n = 4). *** indicate statistically significant difference between control and transfected samples at p<0.001. A typical experimental result is shown. **(B)** MCF-7 cells were seeded in 6-well plates and were transfected with the constructs indicated and were treated with AICAR and/or MTX as indicated. Treatments continued for 6 days and cells were re-transfected daily. Cellular proliferation was quantified by sulphorhodamine B assay. The data is the average of 3 parallel experiments. *, **, *** indicate statistically significant difference between control and treated cells at p<0.05, p<0.01, p<0.001, respectively. #, ##, ### indicate statistically significant difference between mock transfected and transfected samples (same pharmacological treatment, different construct for transfection) at p<0.05, p<0.01, p<0.001, respectively. All abbreviations are in the text. **(C)** The kmplot.com, a freely accessible gene expression database was accessed to analyze the effect of the expression of AMPKα1 and FOXO1 on overall survival in breast cancer patients. We included all patients, also those, where ER status was derived from gene expression data. Overall survival rates were analyzed.

Both short (until day 1) and long term (until day 6) silencing (transfection each day) efficiently reduced the expression of target mRNA (Fig 7A). When these constructs were transfected into MCF-7 cells the proliferation of the cells doubled (Fig 7B). Furthermore, reduced cell proliferation upon AICAR+MTX treatment was abolished by the end of the 6 day treatment (Fig 7B).

### Expression of AMPKα1 and FOXO1 positively correlate with survival in breast cancer

The previously performed experiments nominated AMPK and other mitotropic factors (PGC’s, FOXO1) as possible targets in human breast cancer. To validate this possibility we screened a publicly available cancer gene expression database, (Kaplan-Meier plotter, kmplot.com). When comparing the lowest and the highest expression quartile, higher expression of AMPKα1 and FOXO1 conferred significantly longer survival to the high expression quartile as compared to the lowest expression quartile (Fig 7C). Higher expression of PGC-1α did not confer longer survival (data not shown).

## Conclusions

Breast cancer cells are characterized by Warburg-type metabolism. This is well illustrated by the observations showing that already breast cancer-initiating cells are characterized by reduced mitochondrial activity [13], furthermore, triple negative breast cancer cells display Warburg-type rearrangement [14] and mitochondrial uncoupling [15]. Metabolic reprogramming is crucial in breast cancer cells. For example, mutations in mitochondrial DNA that reduce mitochondrial output or enhance fatty acid synthesis support the development of breast cancer [27,28]. Moreover, the induction of mitochondrial activity by thyroid hormone or docosahexenoic acid in breast cancer cells can accentuate the efficacy of chemotherapy [17,29], while reduced mitochondrial activity or enhanced glycolytic activity correlates with poor prognosis [16,30]. Breast cancer cells reprogram the metabolism of adjacent non-cancerous stromal cells too that is called a reverse-Warburg effect [31] that suggest poor clinical outcome in patients [32]. Importantly, the degradation of AMPK is a key step in turning towards Warburg metabolism [33] that is probably linked to decreases in the mitotropic tone of the energy sensor web and consequently in mitochondrial oxidative metabolism.

Hereby, we provide evidence that the application of the AMPK activator AICAR is capable of potentiating the antiproliferative effects of MTX. In our experiments six days long treatment of MCF-7 cells with AICAR+MTX reduced cell numbers. Importantly, we detected low numbers of PI positive (dead) cells and no change in caspase activity, making decreased cellular proliferation a likely hypothesis over the induction of cell death. Since breast cancer cells are known to depend on Warburg-type rearrangement of metabolism [13–17,27] it was very likely that AMPK activation turned on mitochondrial oxidation and hence induced an anti-Warburg rearrangement of metabolism. Indeed, protracted AICAR+MTX treatment brought about several features of an anti-Warburg metabolic rearrangement, enhances in mitochondrial activity, decreased glycolytic flux. AMPK activation has beneficial effects in *in vivo* experimental lymphoma models [10] or breast cancer cells other than MCF-7 (SKBR-3 and 4T1, as shown here, or MDA-MB-231 [34]). In these models decreased lactate production, glucose uptake, replication rate and enhancement of fatty acid oxidation or oxygen consumption characterize AMPK activation [10,34], similarly to our observations.

By providing the necessary substrates, Warburg metabolism feed forward the cell cycle [5]. The study of Colombo and colleagues [5] showed that the transition through the G1/S checkpoint depends on Warburg-specific enzymes that upregulate glucose and glutamine utilization [5,35]. A study by Beckers and colleagues [34] supports and extends these and our observations by showing that AICAR+MTX treatment reduces the rate of DNA replication. In accordance with that, we observed the accumulation of cells in G1 and G2 suggesting the elongation of the cell cycle through slower transition at the G1/S and G2/M checkpoints both on day 1 and day 6 in AICAR+MTX cells. The appearance of G2/M block was an unexpected finding for us, however, recent reports have shown that the G2 to M transition, similarly to the G1 to S transition, also depends on cellular bioenergetics and glycolytic rate [36,37].

What is the molecular mechanism behind these effects? As expected, upon AICAR treatment we observed AMPK activation. AMPK activation blunts anabolism and induces catabolism, wherein increased mitochondrial oxidation and mitochondrial biogenesis has key role [38,39]. PGC-1α, PGC-1β and FOXO1 are mitotropic transcription factors downstream of AMPK [40–42]. Using an shRNA approach we proved that the overexpression of these master transcription factors are crucial in the AICAR+MTX-mediated inhibition of proliferation and the upregulation of PGCs and FOXO1 upon AMPK activation explains the upregulation of mitochondrial activity. Importantly, an *in silico* approach has already nominated PGCs and NRF1 as key proteins regulating bioenergetics in breast cancer cells [43]. Furthermore, Faubert and colleagues [10] have shown the suppression of the HIF and mTORC1 pathway in experimental lymphoma models upon AICAR treatment that may be implicated in our models too.

Hereby, we provided evidence that the joint application of MTX and AICAR is probably an efficient combination for breast cancer chemotherapy, whereby we suggest a new partner to potentiate the anti-proliferative effects of MTX and provided evidence for the possible applicability of humans. Furthermore, the AICAR+MTX combination exert similar effects in other cancer cells, such as in A431 cells that is a model for human squamous carcinoma [34] pointing out the possible application of that combination in other cancers. Finally, on a wider perspective, it seems that the simultaneous induction of mitochondrial activity and modulation of an independent metabolic pathway may induce synthetic lethality or slow down proliferation in cancers other than breast cancer that can be later exploited in combatting the disease.

## Supporting Information

S1 Data Primary data. (XLSX)

## Abbreviations

ACO2: aconitase-2
AMPK: AMP-activated kinase
AICAR: 5-Aminoimidazole-4-carboxamide 1-β-D-ribofuranoside
ECAR: extracellular acidification rate
IDH2: isocitrate dehydrogenase-2
MTX: methotrexate
OCR: oxygen consumption rate
pACC: phospho-acetylCoA carboxylase
PGC-1: peroxisome proliferator-activated receptor-γ cofactor-1
RT-qPCR: reverse transcription-coupled quantitative PCR
SDS-PAGE: sodium dodecyl sulfate-polyacrylamide gel electrophoresis
SRB: sulphoradamine B
DioC6: 3,3′-dihexyloxacarbocyanine iodide
